# Cellular and Molecular Aspects of Blood Cell–Endothelium Interactions in Vascular Disorders

**DOI:** 10.3390/ijms21155315

**Published:** 2020-07-27

**Authors:** Jean-Luc Wautier, Marie-Paule Wautier

**Affiliations:** Faculté de Médecine, Université Denis Diderot Paris, 75013 Paris, France; mpwautier@hotmail.com

**Keywords:** platelet, red blood cell, leukocyte, endothelial cell, adhesion receptors, vascular cell adhesion molecule (VCAM), Lutheran/basal cell-adhesion molecule (Lu/BCAM), thrombosis, inflammation, vascular occlusion

## Abstract

In physiology and pathophysiology the molecules involved in blood cell–blood cell and blood cell–endothelium interactions have been identified. Platelet aggregation and adhesion to the walls belonging to vessels involve glycoproteins (GP), GP llb and GP llla and the GP Ib–IX–V complex. Red blood cells (RBCs) in normal situations have little interaction with the endothelium. Abnormal adhesion of RBCs was first observed in sickle cell anemia involving vascular cell adhesion molecule (VCAM)-1, α4β1, Lu/BCAM, and intercellular adhesion molecule (ICAM)-4. More recently RBC adhesion was found to be increased in retinal-vein occlusion (RVO) and in polycythemia vera (PV). The molecules which participate in this process are phosphatidylserine and annexin V in RVO, and phosphorylated Lu/BCAM and α5 laminin chain in PV. The additional adhesion in diabetes mellitus occurs due to the glycated RBC band 3 and the advanced glycation end-product receptors. The multiligand receptor binds advanced glycation end products (AGEs) or S100 calgranulins, or β-amyloid peptide. This receptor for advanced glycation end products is known as RAGE. The binding to RAGE-activated endothelial cells leads to an inflammatory reaction and a prothrombotic state via NADPH activation and altered gene expression. RAGE blockade is a potential target for drugs preventing the deleterious consequences of RAGE activation.

## 1. Introduction

Hemorrhagic syndrome was a major cause of death in prehistoric life until the 19th century. Coagulation was first discovered and the fibrin, which was previously called plasmin, identified in the second part of the 19th century. The role of platelets in the first step of bleeding arrest was only recognized in the first part of the twentieth century. In developed countries, beside wars, thrombosis seems to have been another major cause of death in cardiovascular disorders, coronary syndrome, and cerebral ischemia.

The development of molecular biology and cell biology opened a completely new paradigm in thrombosis and hemostasis. After the discovery of the coagulation factors, the molecules present on blood cells and the vessel walls, endothelial cell smooth muscle cell molecules, and sub-endothelium structures were explored. A new step was made when, from the molecules involved in thrombotic and hemostatic processes, we had access to the gene and the gene regulation. The molecules involved in platelet–vessel wall interactions were detected first. Studies of patients with platelet dysfunction such as Glanzmann thrombasthenia, and Bernard–Soulier syndrome, led to the discovery of the role of glycoproteins (GP IIb/IIIa) in terms of their platelet aggregation and GP Ib–IX–V complex with regard to platelet adhesion.

Hemorheological factors were found to have a major function in blood cells and blood cell–vessel wall interactions. Beside plasmatic factors, blood cells participate in the thrombus formation. The role of platelets was first recognized but with the development of hemorheology, the importance of red blood cells in vessel occlusion and clot formation were investigated. Leukocyte functions were characterized during the 20th century. In inflammatory situations leukocytes were shown to contribute to endothelial cell and vessel wall alterations and also to coagulation by the production of tissue factor.

This review demonstrates the important function of red blood cells in vascular disorders. Vaso-occlusion was described in sickle cell diseases as a consequence of red blood cells (RBCs) increasing their adhesion to endothelium. RBC increased adhesion was successively observed in diabetes mellitus, polycythemia vera, and retinal vascular disorders. Polycythemia vera (myeloproliferative disorder) is also complicated by a high frequency of thrombotic complications. The participation of RBCs in this process was found to be also linked to an epigenetic mutation of JAK2 kinase and to a modification of an RBC molecule involved in RBC adhesion. As for hemostasis the study of patients suffering from atherosclerotic disorders or diabetes and ageing patients allowed the discovery of new molecules of the vessel and their functions. The most recent example is the receptor for advanced glycation end products or RAGE.

## 2. Blood Cells and Endothelial Cells

### 2.1. Endothelium

Endothelial cells (ECs) are present in the inner face of the vessels. They have common characteristics but they can be specialized, in particular in the brain, kidney, liver, skin, and coronary vessels. The junctions between the endothelial cells have some differences according to their location in organs. Junctions are dynamic structures. Endothelial cells adhere to one another through junctional structures formed by transmembrane adhesive proteins. Permeability to plasma solutes is controlled, to a considerable extent, by junction permeation. Leukocyte extravasation and infiltration into inflamed areas require finely-regulated opening and closing of cell-to-cell contacts [1,2,3]. ECs participate in the regulation of the vessel tone and synthesize nitrogen oxide (NO) and prostacyclin (PGI2) [4,5]. ECs have receptors involved in blood cell–endothelial cell interactions [6]. ECs synthesize von Willebrand factor which participates in hemostasis [7].

### 2.2. Platelets

During the 70s, antiaggregating agents, such as aspirin, were used as a therapy in coronary disease, stroke, and peripheral vascular disease. Aspirin was shown to act by inhibiting cyclooxygenase, responsible of prostaglandin G2 (PGG2) and prostaglandin H2 (PGH2) PGG2 and PGH2 formation, which are the precursors of thromboxane A2. Anti-platelet drugs were developed and they modified platelet molecules involved in platelet activation and thrombus formation. Platelet glycoproteins (GP), GP llb and GP llla are related to membrane proteins in terms of their structure and functionality; this is also the case for their immunology. These proteins are called cytoadhesins [8] or integrins [9]. They are related to RGD-specific adhesion receptors. This also means that they have a role to play in cell-to-cell interactions. The predominant constituents of the platelet plasma membranes are formed of platelet GP IIb and IIIa (αIIbβ3). These are significant immunogens, which transport the platelet alloantigen. These are also subject to attack from allo- and autoimmune immunologic disorders. Lek (Bak) is found on GP IIb, while PLA occurs on the GP IIIa [10,11]. When we consider patients that have these platelets due to Glanzmann’s thrombasthenia (hereditary blood disorder) [12], we find that the evidence of these genetic markers is significantly limited, or possibly not present at all. This is because they lack GP IIb and IIIa. The GPIb–IX–V complex which is missing in Bernard–Soulier syndrome is the major receptor for adhesion to collagen of the sub endothelium [13]. Von Willebrand factor, when absent, is associated with a congenital hemorrhagic syndrome and is implicated in the platelet adhesion mechanism [7]. The concept that platelets may interact directly with ECs conditioned through an inflammatory environment has received increasing attention. Normally ECs present an antithrombotic surface for flowing blood by releasing nitric oxide (NO) and prostacyclin (PGI2) and through expression of CD39. Signaling into the platelet by PGI2 and NO maintains high levels of cyclic nucleotides within the platelet cytoplasm, thereby antagonizing the known pathways of platelet activation [14].

### 2.3. Leukocytes

**Figure 1:**
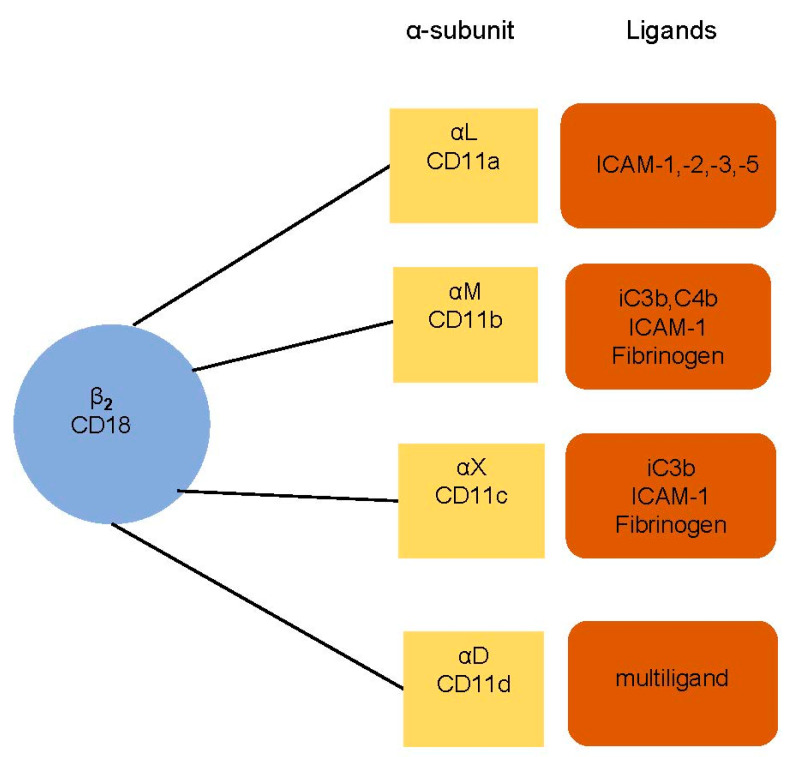
Leukocyte integrins and their ligands. The subunit CD18 (β2 integrins) is associated with α subunit (αL CD11a, αM CD11b, αX CD11c, and αD CD11d). The integrins bind to a specific ligand—intercellular adhesion molecules (ICAMs), fibrinogen, or complement components (iC3b, C4b, c3b). CD11d is a multiligand receptor.

The adhesion of leukocytes is able to be significantly extended when there is the presence of inflammation, infection, or heart disease [14]. The leukocyte–endothelium interaction and its molecular foundations have been the subject of considerable study in recent years [15,16,17]. Monoclonal antibodies enable us to understand the leukocyte adhesion molecule (LeuCAM). This is mediated by integrins β2 (CD18), α subunits (CD11a, CD11b, CD11c, CD11d) on leukocytes, and intercellular adhesion molecules (ICAMs) and vascular cell adhesion molecules (VCAMs) on endothelium. There is an illustration in Figure 1.

The molecule interaction participates in rolling, stable adhesion, and extravasation of leukocytes. In addition to this, other leukocyte structures are connected to this process [18]. These include fibronectin receptors. Researchers have announced that there are particular structures which are connected to the endothelial cells [19]. These cells also express the intercellular adhesion molecules (ICAMs) when there is no stimulation whatsoever. However, E-selectin, and the vascular cell adhesion molecules (VCAMs) can only be located on endothelial cells which have been activated. Cytokines such as TNF-α, IL1-β, and IFN-γ, are able to induce the synthesis and expression of E-selectin and VCAMs. They can also elevate ICAMs and the human leukocyte antigens (HLAs) diabetic retinopathy (DR), DP, and DQ. Interactions between the selectin family and their respective ligands will mediate rolling adhesion of leukocytes on EC under flow conditions [14,20,21] This first step of leukocyte adhesion is then followed by interactions between members of the CD18 family with their respective ligands in the immunoglobulin family that mediate firm adhesion/arrest on leukocytes on the ECs. The leukocyte adhesion mediated by P-selectin could occur when inflammatory mediators such as histamine or thrombin activate ECs. This activation redistributes P-selectin from its storage granules to the endothelial surface. On the other hand, the expression of E-selectin on the endothelial cell surface is indeed induced only by stimulation of ECs by inflammatory cytokines (e.g., IL-1 and TNF-α). ICAMS are also major adhesion molecules that arrest circulating leukocytes on ECs.

Acute respiratory distress syndrome (ARDS), hypercholesterolemia, and diabetes mellitus are all involved in the elevation of leukocyte adhesion. This then results in vascular damage. Platelet–leukocyte aggregates occur in atherosclerosis and atherothrombotic disease. This means that researchers need to focus on modifying the leukocyte–endothelial cell in terms of anti-thrombotic and anti-atherosclerotic treatments.

Macrophage activation in atherosclerotic lesions is associated with the local release of inflammatory cytokines and reactive oxygen intermediates. During myocardial infarction, cardiomyocytes, tissue-resident macrophages, and fibroblasts passively release damage-associated molecular patterns, such as ATP and hyaluronic acid through the breakdown of the extracellular matrix. This activates local macrophages and neutrophils and stimulates a proinflammatory immune response via chemokines, cytokines, and vascular adhesion molecules through intracellular mitogen-activated protein kinases (MAPK) and NF-κB signaling [22].

SARS-CoV-2 binding to the angiotensin-converting enzyme receptor present on endothelial cells may facilitate the interaction with leukocytes, enhancing the expression of adhesion receptors. This inflammatory reaction producing interleukin-6 (IL6), macrophage chemoattractant protein-1 (MCP1), and tissue factor, can lead to thrombosis and promote apoptosis.

### 2.4. Red Blood Cells

#### 2.4.1. Red Blood Cell Adhesion in Sickle Cell Anemia

Sickle cell disease (SCD) causes abnormalities in terms of function. This hematological disease showed how the RBC structure creates issues. The sickling is observed when the oxygen partial pressure is lowered, hemoglobin S polymerized, and red blood cell deformability reduced. Hebbel [23] argued that patients who suffer from sickle cell disease have issues in the vascular endothelium. An experimental model with cultured endothelial cells and red blood cells labelled with chromium-51 allows us to see the effects of this condition. During vaso-occlusive episodes these issues are elevated [24].

**Figure 2:**
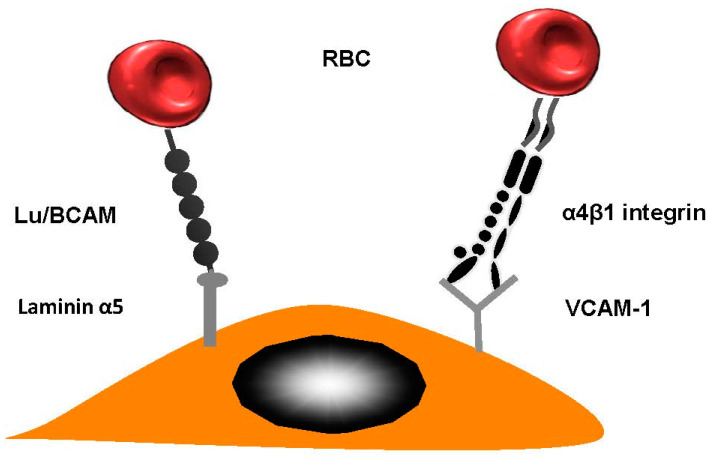
Red blood cell (RBC) adhesion in sickle cell diseases. Lutheran Lu/BCAM contains a particular protein which is able to forge a connection with laminin α5. The latter occurs in the sub-endothelium; however, it can also occur on the apical side endothelial cell. VLA-4 (α4β1 integrin) is a molecule produced by reticulocytes; this had a strong sticky quality.

This sticking of RBCs to vascular walls facilitates the creation of HbS polymers. This is a result of restricting the movement of cells through the capillaries. This then has a chain reaction and causes vaso-occlusion. Many research studies have confirmed the fact that abnormal cell adhesion limits micro vessels and traps sickle cells within postcapillary venules. The movement of RBCs could be reduced in patients suffering from sickle cell disease by ensuring they remain in contact with the vascular wall by using erythroid adhesion molecules and endothelial cell proteins. Researchers have written about the interactions that occur between HbSS RBCs and the endothelial vascular wall. When α4β1 integrin (VLA-4) occurs on newly-formed moving reticulocytes, it plays a significant role as they enable RBC connection to vascular cell adhesion molecule (VCAM-1), amongst other substances [25] (see Figure 2 for more details). HbSS reticulocytes are bound to the endothelial cells using thrombospondin (TSP) which also connects their CD36 molecules [26]. That said, this does not reduce the significant impact of SCD in terms of patients who lack CD36 [27].

#### 2.4.2. Red Blood Cell Adhesion in Polycythemia Vera.

In 1892, polycythemia vera (PV) was first discovered. It was found in a person suffering from thrombosis.

It can be diagnosed through bone marrow culture, determination of red cell mass, and JAK2 mutation. The incidence of thrombotic events is very high in patients with PV. According to different studies it varies between 12% and 39% at the time of diagnosis. Recurrent thrombosis varies between 31% on the venous side and 67.6% on the arterial side. The risk of suffering from this condition is higher in patients with a high level of JAK2 V617F allele mutation [28]. JAK2 mutation occurs in more than 98% of people with PV and about 30% of patients with thrombocythemia. In patients with thrombosis of hepatic, portal, or mesenteric veins, this type of mutation was found in 34% and 45% in patients with Budd–Chiari syndrome [29].

**Figure 3:**
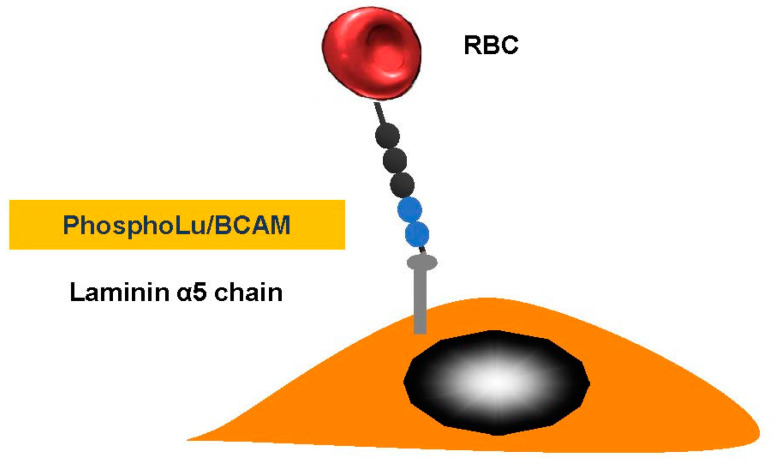
Polycythemia vera. Erythrocyte-phosphorylated Lu/BCAM can be found in people with polycythemia vera, as a consequence of a JAK2 V617 F mutation. This then affects the endothelial cell laminin α5, which occurs in the sub-endothelium and the apical-side endothelial cell. The Lu/BCAM cytoplasmic domain is affected by a cell adhesion to the laminin protein within a spectrin-based skeleton.

We discovered that red blood cell (RBC) adhesion of patients with PV was increased and we explored the molecular basis of this abnormality [30] (Figure 3). Our in vitro results with PV strongly suggest that RBC–endothelium interaction may have deleterious consequences. PV RBC adhesion activates endothelial cells and stimulates vascular cell adhesion molecule expression favoring leukocyte adhesion.

JAK2 mutations found in people suffering from PV [31,32] may create myeloproliferation. They can also cause greater numbers of blood cells and a greater likelihood of functional abnormalities. Lu/BCAM is expressed after band 3 during erythropoiesis and it first appears on early orthochromatic erythroblasts [33,34]. The fact that the disease could be easily cloned might give a reason for why its excessive adhesiveness is not limited to just reticulocytes but also involves additional moving RBCs. Patients who suffer from PV are often subject to thrombotic complications and cerebrovascular issues. Post-sinusoidal obstruction can often be found beyond the liver in the hepatic veins in patients suffering from Budd–Chiari syndrome. The variable response in flow could give an indication of why thrombosis occurs systematically.

#### 2.4.3. Red Blood Cell Adhesion in Retinal-Vein Occlusion

**Figure 4:**
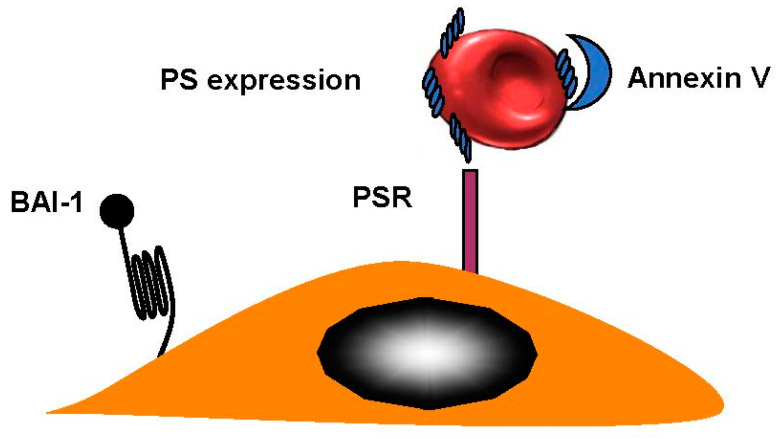
Retinal-vein occlusion. RBCs from patients with retinal-vein occlusion have an enhanced phosphatidylserine (PS) expression and adhere to the endothelium. Brain-specific angiogenesis inhibitor (BAI-1) and phosphatidylserine receptor (PSR), are potential receptors. Blockade of PS RBCs by annexin V or PS receptors on endothelial cells by anti PSR, inhibited adhesion indicating that the couple PS–PSR is responsible for increased adhesion of RBCs from patients with CRVO.

Sight loss in elderly people can be caused by retinal-vein occlusion (RVO). It is the second-most-common reason for loss of sight; diabetic retinopathy is the primary cause of reduced vision. There are three sections in the anatomic classification—hemi-retinal-vein occlusion (HRVO), central-retinal-vein occlusion (CRVO), and branch-retinal-vein occlusion (BRVO). A retinal hemorrhage can indicate RVO, and so can venous dilation, which can sometimes occur with retinal swelling in a particular area. This can be diffuse for CRVO, as part of a hemiretina for HRVO or, in the case of BRVO, in connection with a retinal quadrant. BRVO is the least-serious form of RVO, which is probably due to the fact that it is found at an arteriovenous crossing [35]. The worst is central-retinal-vein occlusion (CRVO) and there is very little known about this condition. RBCs from patients with CRVO were more adherent when they express phosphatidylserine and bound to the phosphatidylserine (PS) receptor. Annexin V binding to PS RBCs inhibited adhesion to endothelial cells [36] (Figure 4). BRVO occurs at 0.6%, while CRVO is limited to 0.1%. BRVO is related to hypertension [37]. Central-retinal-artery occlusion (CRAO) is connected to eye strokes. This can appear in one in every 100,000 patients [38].

## 3. RBC Adhesion and Molecular Alterations in Diabetes Mellitus

### 3.1. Red Blood Cell Adhesion in Diabetes Mellitus

Current research indicates that hemorheological factors have a role to play in many thrombotic diseases. In addition, diabetes mellitus is connected with an increased incidence of microvascular and atherosclerotic disease. It is also important to note that erythrocyte abnormalities can be found in people suffering from diabetes. Studies have found that these issues are connected with a larger amount of whole-blood and plasma viscosity. However, they have also found that it had a negative impact on the formation of erythrocytes. Washed erythrocytes adhered more strongly to cultured endothelial cells in diabetic patients [39]. The extent of red-cell adhesion is connected to severity of diabetic vascular disease; however, it does not appear that this changes with age or the duration of the condition [40]. People suffering from diabetes can also have a non-enzymatic glycosylation with regard to quite a few proteins. This is also the case for red-cell-membrane glycoproteins, which may be a reason for abnormal red cells.

### 3.2. Advanced Glycation End Products (AGE)

**Figure 5:**
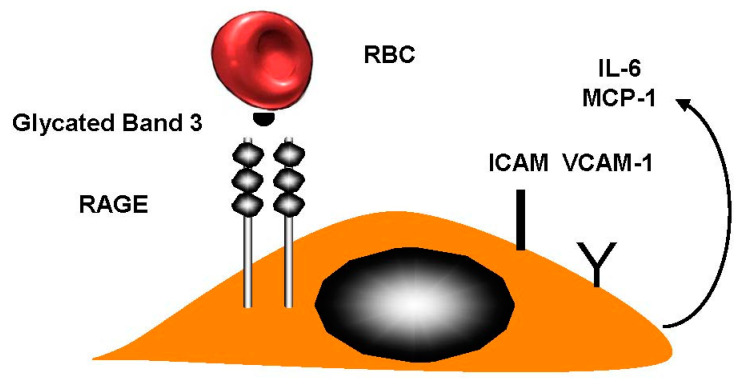
Diabetes mellitus. When people suffer from diabetes the RBC-band-3 protein is also likely to be glycated (AGE). The binding to the endothelial cell receptor for advanced glycation end products (RAGE) activates ECs, inducing tissue factor (TF) production, intercellular cell adhesion molecule-1 (ICAM-1) and vascular cell adhesion molecule (VCAM)-1 expression. The activation induced interleukin-6 (IL-6), vascular endothelial growth factor (VEGF), and macrophage chemoattractant protein-1 (MCP-1) release.

L.C. Maillard was the first person to examine and explain melanoidin formation in 1912 [41]. This is an interaction between free amino protein groups, which are predominately formed of lysine, arginine, and carbohydrates. Then, Amadori discovered the Amadori and Heyns rearrangement. However, there is still a lack of knowledge with regard to Maillard products. HbA1c was first used as a biomarker; this is a result of non-enzymatic glycosylation (glycation) [42]. There are two locations where hemoglobin is glycated. Firstly, it occurs on the valine residue of the N-terminal β chains. This is located by the α and β chains of the ε amino group. Secondly, it can be found at the α chains N-termini [43]. The level of HbA1c is used to adapt antidiabetic treatment, insulin, or oral antidiabetic therapy. RBC-band-3 membrane protein is glycated in diabetic patients. Glycated RBC band 3 binds to the receptor for advanced glycation end products (RAGE) and is responsible for the increased RBC adhesion observed in diabetes mellitus (Figure 5).

Glycoxidation products occur due to glucose deterioration. Additionally, glyoxal and methylglyoxal (MG) create AGE [44,45]. This can be situated in extracellular or intracellular areas [46]. Major AGE are *N*-epsilon (carboxymethyl)lysine (CML) [47], MG-derived AGE and pentosidine [48].

AGE occur in dietary products and they are changed within the body before the body expels them through the kidneys [49]. AGE are also endogenous, they can be partly degraded by glyoxalase and eliminated by the kidney, but they accumulated with aging [50].

### 3.3. AGE Receptors

RAGE was initially believed to contain two polypeptides, one a lactoferrin-like component (80 KDa) and another a polypeptide (35 KDa) [51]. AGE are extremely damaging to the body. It is also important to note that RAGE has the ability to connect a considerable amount of endogenous molecules and amphoterin c, as well as β-amyloid peptide and S100 calgranulins. RAGE, a member of the immunoglobulin (Ig) superfamily, has three Ig-like domains—an extracellular part with one variable (V) and two constants (C1 and C2), a single transmembrane domain, and one short cytosolic domain [52]. Researchers have attempted to examine the RAGE polymorphism [53].

**Figure 6:**
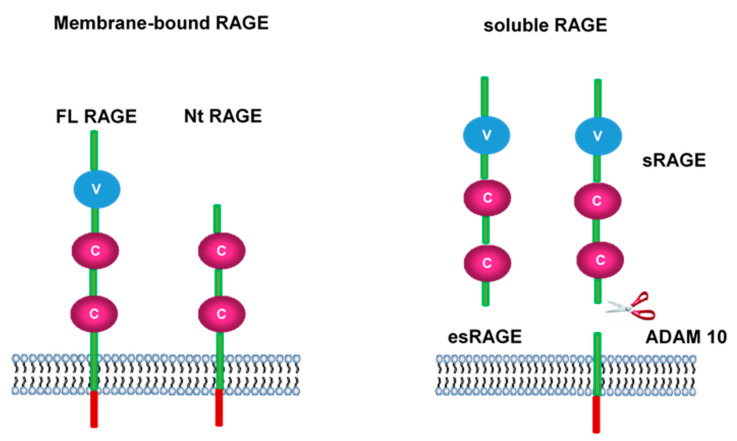
RAGE isoforms. The receptor for AGE (RAGE) can be produced as a complete molecule (full length, FL-RAGE) or as a dominant negative (Nt RAGE) which is a non-efficient receptor. Soluble forms of RAGE can result from an incomplete transduction of the gene (esRAGE) or from a proteolytic reaction by ADAM 10 metalloproteinase.

When RAGE transcripts are separated they can create up to 20 different forms [54]. However, it is important to note that there are only three types of RAGE that can be found in endothelial cells. These include N-truncated (Nt-RAGE), full length (FL-RAGE), and endogenous secretory (esRAGE). It is possible to produce soluble RAGE (sRAGE) via a process known as FL-RAGE proteolysis (Figure 6). RAGE occurs at minimal levels elsewhere in the body.

Methylglyoxal human serum albumin (MG-HSA) and *N*-(carboxymethyl)lysine human serum albumin (CML-HSA) connect to the receptor and have an effect on RAGE isoform transcripts. There are three forms of RAGE found in these types of cells, which are affected by MG-HSA. However, CML-HSA just occurs in FL- and Nt-RAGE isoforms. It was discovered that both MG-HSA and CML-HSA have a role to play in terms of RAGE expression. Nevertheless, MG-HSA enhances esRAGE expression, potentially implicating a negative feedback loop because soluble RAGE generated may act as a decoy intercepting the interaction of ligands with cell-surface RAGE, thereby limiting RAGE mediated cellular activation. This could restrict the production of RAGE-mediated cellular activation [55]. It is possible that RAGE isoform expression might affect the vascular bed, which could leave it open to damage from RAGE ligands. FL-RAGE and ligand interaction can speed up the effects of disease. That said, they can also restrict positive actions, as they do not have cellular signaling domains.

### 3.4. AGE and RAGE

Researchers have been studying the link between glycated proteins and vascular disease for many years [56]. The work has concentrated on animals as well as glycated proteins in nephropathy. Anti-RAGE was tested in rats with diabetes and it was found that the antibody limited the increased permeability of the vascular wall [57]. AGE are also connected to renal failure and inflammation. *N*- epsilon (carboxymethyl) lysine (CML)-adducts react to RAGE and affect signal transduction pathways, leading to expression of certain genes.

The receptor RAGE affects genes [51,58]. RAGE cytoplasmic domain (ctRAGE) interacts with diaphenous-1 (DIAPH1), a formin molecule, both in vitro and in vivo. When ctRAGE encounters DIAPH1 it impacts a number of key consequences, including modulation of the actin cytoskeleton and cellular migration, activation of the Rho GTPases, and generation of reactive oxygen intermediates.

There is a type of RAGE that is lacking the intracellular domain, which is able to connect to a ligand. It also has a “dominant negative” impact on CML-adducts. CML binding to RAGE activates NF-κB, however this does not really occur in DN-RAGE-transfected cells.

**Figure 7:**
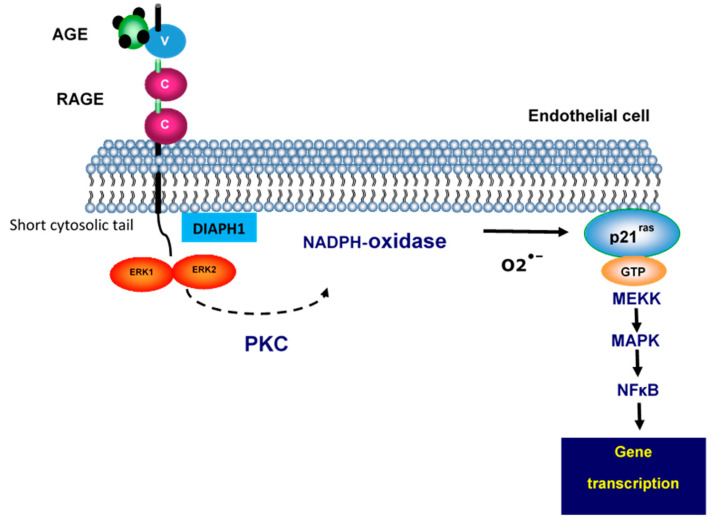
Binding of AGE to the endothelial cell receptor, RAGE. This is followed by a series of reactions involving diaphenous-1 (DIAPH 1), ERK1, ERK2, and protein kinase C, which lead to NADPH oxidase activation and the formation of reactive oxygen intermediates. Through a pathway including the MAP kinase, NFΚB becomes activated and induces gene transcription.

The interaction of β_2_Microglobulin AGE (AGE-β_2_M) or AGE formed on the surface of red blood cells or AGE immune-isolated from the blood of patients suffering from diabetes or renal failure with RAGE, generated reactive oxygen intermediates (ROI) (Figure 7) [59].

**Figure 8:**
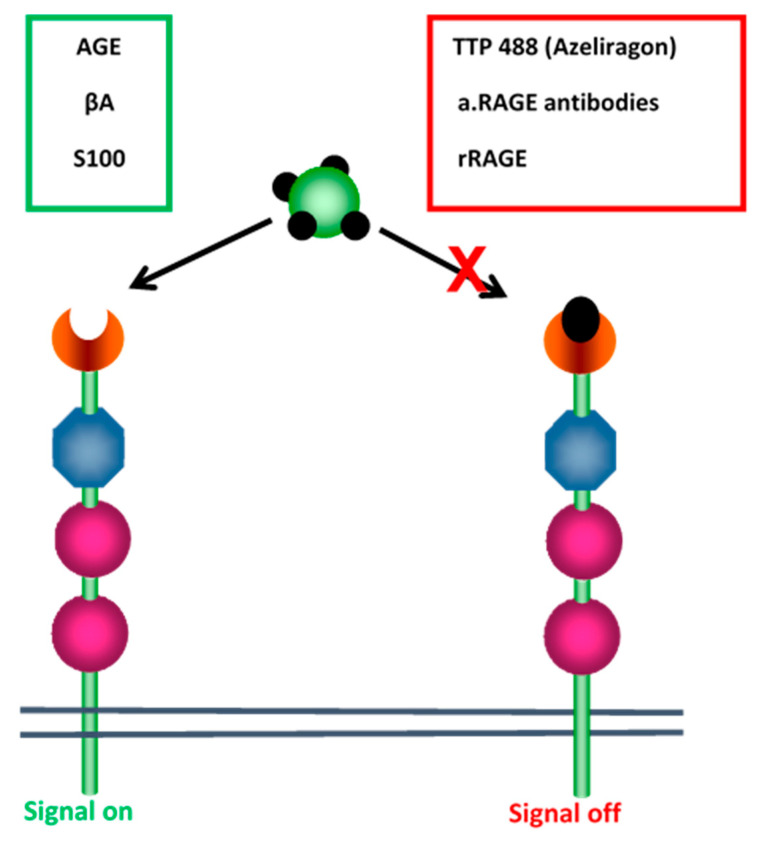
Blockade of RAGE activation. RAGE molecule has an extracellular part with two constant domains (pink circles) and one variable domain (blue octagon). The binding of AGE, βAmyloid protein and S100 calgranulins to RAGE region (brown) can be inhibited by anti-RAGE antibodies or a synthetic peptide TTP488. However, recombinant RAGE is able to bind the AGE ligands competing with the cellular RAGE. The blockade resulted in the absence of signal transduction in endothelial cells.

RAGE affects ERK and it is able to modify both its substrate and its subcellular location. It can also engage with intracellular signals through NF-κB. Therefore, researchers are currently attempting to determine which molecular mechanism affects it. Also, there is an issue in terms of the ROI as this is important for the cellular ligation of RAGE via AGE. Following the activation of NADPH oxidase based on the AGE–RAGE interaction, it was found that ROI appeared. This set off a chain of events which then had an impact on the gene expression within a given cellular microenvironment. Anti-RAGE anti-bodies are able to interrupt the damaging effects of RAGE; this is also the case for RAGE analogs TTP488 (azeliragon) and recombinant rRAGE (Figure 8).

## 4. Vascular Complications in Diabetes Mellitus

### 4.1. Retinal Vascular Lesions

Diabetic macular edema and proliferative diabetic retinopathy (PDR) are primarily responsible for sight loss in people with diabetes. It is important to note that macular edema affects the inner blood retinal barrier [35]. It is possible that when someone suffers from diabetic retinopathy (DR) they can also develop diabetic maculopathy. This can occur in both the non-proliferative and the proliferative stage. When DR was examined, it was clear that the retinal capillaries underwent certain changes, such as a thickening in the basement membrane, reduced pericytes, and greater permeability and vascular dysfunction. These changes are both morphological and functional. Type 1 diabetes also comes with a greater likelihood of suffering from PDR than type 2. That said, the second type of diabetes has an increased risk of diabetic macular edema.

The retinal alterations result in two components. When macromolecules (e.g., lipoproteins) leak they cause macular edemas because they damage the retinal layers. There are also issues that result from micro-thrombosis, which can lead to the closure of capillaries. The retina can be affected by hyperglycemia in various ways this can lead to biochemical alterations. This can, in turn, lead to a variety of conditions including oxidative stress, protein kinase C, and AGE [60]. When the capillaries are closed it can cause non-perfused hypoxemic retinal sections (ischemic retinopathy). This then leads to the expression of vascular endothelial growth factor (VEGF), which then creates additional vessels (proliferative retinopathy) [42]. Panoramic fundus fluorescein angiography and 360-degree fluorescein gonioangiography can be used to examine angle neovascularization and retinal ischemia in patients suffering from PDR. When there is retinal non-perfusion in the mid periphery, it can lead to capillary occlusion in the radial peripapillary capillaries. There was also another issue, in that angle neovascularization can be caused by temporal raphe and an optic disk [61]. In addition, VEGF is able to permeate the vessels and it is also pro-angiogenic. VEGF165 or VEGF-A are the types of this condition that are often found in people. VEGF-A operates on endothelial cells. In order to do this, it uses two receptor forms—the VEGF- receptor 1 (VEGF-R1 (Flt-1)) and the VEGF receptor -2 (VEGFR-2 (Flk- 1/KDR)). The latter can increase the risk of angiogenesis [62]. Chronic hyperglycemia has a significant part to play with regard to diabetic retinopathy. This can occur in both forms of diabetes, which has been proved by the Diabetes Control and Complications Trial [63]. Evaluating glycated hemoglobin is an essential part of treatment for this condition. It has also been proved that there is a significant positive correlation when the hyperglycemia rises due to increased glycated hemoglobin (HbA1c). This also has an effect on retinal lesions which will become larger, more frequent, and more serious [64]. It has been clear for a long time that AGE are involved in retinopathy. People suffering from diabetes often also suffer from proliferative retinopathy associated with greater amounts of pentosidine in their skin [65]. This is also the case with 2-(2-fuoryl)-4(5)-(2-furanyl)-1H-imidazole (FFI), *N*-epsilon (carboxymethyl) lysine (CML), and fluorescence [66]. Glycation can also be found in RBC intracellular and membrane proteins. This occurs in spectrin, as well as band-3 transmembrane protein and band 4–1 [67]. This reduces RBC deformability and increases adhesion to endothelium [39,58]. The sRAGE and anti-AGE antibodies limit the adhesion to endothelium when incubated with diabetic RBC. High glucose concentrations have extremely negative effects on capillary pericytes. However, this can be prevented by the introduction of aminoguanidine in animal experiments. Endothelial cells (ECs) and pericytes need to be protected from damage as they have a vital role to play in DR [68]. We need to spend more time researching pericyte and EC dysfunction as we need to know how to prevent their deterioration in order to improve retinal vascular regulation. There have been previous examinations of the negative impact of AGE in terms of retinal capillary pericytes and endothelial cells. It was found that they can be limited by RAGE antibodies, which shows an incredibly promising potential for future treatments. There have been studies into VEGF, as it is believed that it can affect vascular dysfunction due to pseudo-hypoxemic alterations. This, however, can be restricted by deploying VEGF antibodies.

### 4.2. Soluble RAGE (sRAGE)

Retinopathy and glomerulosclerosis often occur as a result of diabetes. When RAGE is activated, it can lead to greater oxidative stress, permeability, and inflammatory issues in the vessels. It is also worth noting that sRAGE can be created by splicing its gene transcript. It is possible that sRAGE has the potential to prevent AGE-related damage and that it can also provide an indication of the risk of dying from cardiovascular disease. It has been noted that *N*-epsilon (carboxymethyl)lysine (CML)-protein occurs to a larger degree in people with diabetes that also have microvascular issues. However, there is considerably less sRAGE in people who have retinopathy and nephropathy at the same time. People with diabetes have elevated levels of CML-protein but it occurs most frequently in people with microvascular problems [69]. This indicates that sRAGE cannot decrease the CML-protein. It is worth noting that this leads AGE to connect to RAGE, which, in turn, causes endothelial problems, which lead to microvascular issues. However, when there is a larger amount of sRAGE and the CML-protein levels are reduced, this does not occur. This means that sRAGE could ensure appropriate conditions in the vessels. Indeed, when we consider type 2 diabetes, it is clear that when there is less sRAGE present, the patient is more likely to die from heart complications. However, in Type 1 diabetes, reduced sRAGE causes more serious retinopathy and thicker vessels [70]. In type 2 diabetes, there is a correlation between sRAGE and serum levels of macrophage colony-stimulating factor (MCSF) as well as tumor necrosis factor alpha (TNF-alpha). Therefore, it is no surprise that it is considered to indicate inflammation in the vessels [71]. Using sRAGE to restrict RAGE also had an effect on atherosclerotic lesions. This is because it prevents stress on the body [72]. Certain drugs, e.g., angiotensin-converting enzyme (ACE) inhibitors and rosiglitazone, modify RAGE and lead to the increased prevalence of sRAGE [73]. This means that more research needs to be undertaken with regard to sRAGE as it could have beneficial effects for AGE-related vascular disease.

sRAGE levels do not really vary over the course of a few years. That said, there are significant variations in terms of the amount of sRAGE which is dependent on the sufferer’s ethnicity. It is absolutely vital that this is factored into calculations. It was believed that RAGE gene polymorphisms have a connection to sRAGE but this has yet to be proved. There is evidence to suggest that older people have greater levels of this substance than younger adults. Research has proved, however, that people who suffer from type 2 diabetes are more likely to have significantly higher incidence of microangiopathy. There have also been experiments that examined macroangiopathy and cardiovascular mortality, and their connection to sRAGE. The connection to vascular risk is highly debated. There was a piece of research which examined diabetes (hazard ratio 1.64 (95% CI 1.10–2.44)), coronary heart disease (1.82 (1.17–2.84)), and mortality (1.72 (1.11–2.64)). This study found that all of these severe conditions were connected to the sRAGE plasma level [74].

Research based on plasmatic sRAGE has not considered that esRAGE is different to sRAGE. This has led to some issues with their results. There is also the question of ethnic variations in terms of the amount of sRAGE. This is essential to factor in when considering the relevant risks involved. It is important to factor these markers in order to correctly assess the situation.

### 4.3. Preventing AGE

The most effective drugs are based on insulin and oral anti-diabetic drugs. Investigations are ongoing into SGLT1 and SGLT2 inhibitors (glifozins) and various other polytherapies based on glifozins and gliptins [75,76]. Researchers have examined the conjunction of insulin with glifozins, which has indicated positive reactions on blood pressure and obesity [77]. Dipeptidyl peptidase-4 (DPP4)-inhibitors are also responsible for improving glycemic control and are known to engage with the AGE–RAGE axis. Researchers have noted that rats that lack DPP4 activity [78] have limited diabetic nephropathy.

In addition, most research has unsuccessfully attempted to address the issues with the AGE–RAGE axis. However, currently tests are ongoing into an AGE inhibitor’s capacity to produce pyridoxamine dihydrochloride (pyridorin©) [79].

## 5. Conclusions

This review emphasized how the interaction between blood cells and endothelium is important, but the system is more complex and involves blood cells, plasmatic components, and vessel wall reactions. Obviously, the therapeutic approaches also target plasmatic abnormalities and the correction of dyslipidemia, hypercoagulability, and various metabolic disorders. In addition, infections and immunological dysfunctions are important participants in vascular abnormalities and can be counteracted by specific treatment and prevention.

Understanding molecular mechanisms of blood cell–endothelial cell interactions will allow us to design future treatments with better outcomes by using humanized specific monoclonal antibodies, peptides, and gene transcription modulators.
